# Long non-coding RNA DDX11-AS1 promotes esophageal carcinoma cell proliferation and migration through regulating the miR-514b-3p/RBX1 axis

**DOI:** 10.1080/21655979.2021.1940617

**Published:** 2021-07-19

**Authors:** Chao Wu, Zhibin Wang, Xuetao Tian, Jianqiang Wang, Yuesong Zhang, Biao Wu

**Affiliations:** a Department of Anorectal Surgery, Huazhong University of Science and Technology, Wuhan, Hubei, China; b Department of Jiangbei Hospital, Huazhong University of Science and Technology, Wuhan, Hubei, China; c Department of Oncology, the Fifth Hospital of Wuhan, Wuhan, Hubei, China; d Department of Thoracic Surgery, Huazhong University of Science and Technology, Wuhan, Hubei, China; e Department of Hepatobiliary Hernia and Vascular Surgery, Huazhong University of Science and Technology, Wuhan, Zhejiang, China; f Department of Thoracic Surgery, Ningbo Yinzhou NO.2 Hospital, Ningbo, China

**Keywords:** Lnc rna ddx11-as1, esophageal carcinoma, miR-514b-3p, rbx1

## Abstract

Esophageal carcinoma (ESCA) is one of the most aggressive malignancies with extremely high morbidity and mortality. At present, limited advancement in ESCA treatment has achieved. Therefore, it is urgent to explore the pathogenesis and progression mechanism of ESCA to provide the basis for the formulation of novel therapeutic strategies. Previous studies have found that long non-coding RNA (lncRNA) DDX11-AS1 expression enhances the paclitaxel resistance of ESCA cells. However, the mechanisms underlying the drug resistance conferred by lncRNA DDX11-AS1 in ESCA remains to be elucidated. Our research aims to clarify the role and mechanism of lncRNA DDX11-AS1 in regulating the progression of ESCA. We found that the expression of lncRNA DDX11-AS1 in ESCA tissues and cell lines was significantly upregulated. Subsequently, silencing lncRNA DDX11-AS1 significantly inhibited the proliferation, migration and invasion of ESCA cells, and induced the level of cell apoptosis. In terms of mechanism, our data showed that miR-514b-3p/RING box protein 1 (RBX1) axis played a crucial role in the oncogenic function of lncRNA DDX11-AS1. LncRNA DDX11-AS1 expression impaired the inhibitory function of miR-514b-3p on RBX1 through sponging effect. Taken together, our data support the notion that lncRNA DDX11-AS1 promotes the progression of ESCA through miR-514b-3p/RBX1 axis. Our research uncovers the novel regulatory role of lncRNA DDX11-AS1 in ESCA and lays a theoretical basis for developing novel treatment strategy of ESCA.

## Graphical abstract

**Figure:**
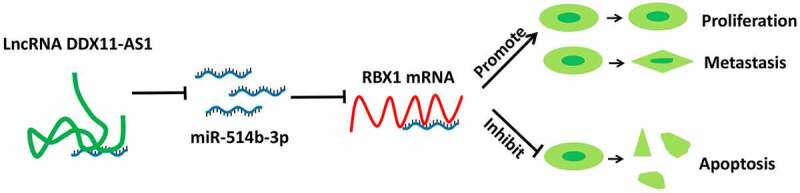

## Introduction

Esophageal carcinoma (ESCA) is a type of malignant tumor characterized by high level of aggression and high mortality rate [1]. ESCA is divided into two subtypes. The most common type occurs in the upper and middle part of the esophagus and is called esophageal squamous cell carcinoma; The less common type occurs in the lower part of the esophagus connected to the stomach and is called esophageal adenocarcinoma [2,3]. Many patients with ESCA were diagnosed with metastasis before receiving treatment, which accounts for an overall low survival rate of patients [4]. The current treatment methods for ESCA include surgery, immunotherapy and systemic chemotherapy [5–7]. Accumulating evidence suggests a crucial role of biomarkers for the diagnosis and treatment of ESCA [8]. Therefore, exploring the mechanism of the origin and progression of ESCA can identify new targets for developing novel therapeutic strategies.

Long non-coding RNA (lncRNA) and microRNA (miRNA) are non-coding RNAs transcribed from the genome [9]. The length of lncRNA is greater than 200 nucleotides, and it is involved in wide range of biological processes of cancer cells, such as proliferation, invasion and immune escape [10,11]. The length of miRNA is between 20–22 nucleotides, and it is implicated in cancer progression through the regulation of a series of cancer-related genes [12]. Recent studies have found that lncRNA serves as a competitive endogenous RNA (ceRNA) of miRNA, thereby reducing the inhibitory effect of miRNA on downstream target genes [13]. For example, increased expression of lncRNA JPX in lung cancer tissues promotes the progression of cancer by inhibiting miR-145, which in turn increases the expression of CCND2 [14]. Overexpression of LncRNA OIP5-AS1 promotes the development of pancreatic ductal adenocarcinoma, and enhances the malignant phenotype by controlling miR-429/Forkhead Box D1 (FOXD1)/extracellular signal-regulated kinase (ERK) axis [15]. In addition, LncRNA LINC01614 can also regulate the expression of a disintegrin A metalloprotease 12 (ADAM12) by sponging miR-383 to sustain the the development of glioma [16]. Therefore, it is crucial to clarify the influence of lncRNA on cancer progression and the regulation of miRNAs in ESCA.

RING box protein-1 (RBX1)/regulator of Cullins1 (ROC1) functions as an important part of Skp1-Cul1-F-box-protein (SCF) ubiquitin ligases [17], and is widely expressed in human heart, kidney, skeletal muscle and other tissues [18]. Accumulating evidence has revealed that RBX1 is highly expressed in many types tumor cells. In hepatocellular carcinoma, RBX1 promotes tumor cell growth and the development of cell malignant phenotypes [19,20]. In non-muscle invasive bladder cancers (NMIBCs), RBX1 is an independent prognostic marker, which is closely associated with tumor size, stage and patient survival [21]. RBX1 also functions to promote the proliferation of gastric cancer cells, and RBX1 expression correlates with a poor prognosis [22,23]. However, whether and how RBX1 regulates the progression of ESCA remains to be further investigated.

In this study, we analyzed the expression difference of lncRNA DDX11-AS1 in ESCA tissues and normal tissues through the TCGA and GEPIA database, and further validated the results in 76 pairs of ESCA tissues and adjacent normal tissues as well as in ESCA cells lines. Our data demonstrated that the expression of lncRNA DDX11-AS1 was higher in ESCA tumor tissues and cancer cell lines, indicating an oncogenic role of lnc RNA DDX11-AS1 in accelerating the progression of ESCA. We therefore sought to clarify the regulatory mechanism of lncRNA DDX11-AS1 in ESCA. Our data show that expression level of lncRNA DDX11-AS1 was correlated with the survival of ESCA patients. Silencing lncRNA DDX11-AS1 impaired the cell proliferation, invasion and induced apoptosis by interfering with miR-514b-3p in ESCA cells. LncRNA DDX11-AS1 expression impaired the inhibitory function of miR-514b-3p on RBX1 through sponging effect. Collectively, our data revealed a novel role of miR-514b-3p/RBX1 axis in ESCA progression and lays a theoretical basis for developing novel treatment strategy of ESCA.

## Materials and methods

### Tissue samples

A total of 76 pairs of ESCA tissues and adjacent normal tissues were collected from Ningbo Yinzhou NO.2 Hospital from March 2017 to June 2019. The age of the patients ranged from 35 to 70 years old. Patients who provided the tumor tissues were informed of the facts and signed an informed consent form. The inclusion criteria were as below: 1) all patients were diagnosed with ESCA and confirmed by pathological staining; 2) all patients had not received radiotherapy or chemotherapy before surgical resection; 3) all patients had complete clinical diagnosis materials and medical records. The exclusion criteria were as below: patients who suffered from other malignant tumor diseases or organic diseases or received treatment. The other conditions of the recruited patients were showed as below:

1) Gender: 50 males and 26 females; 2) Age: 35 to 70 years old; 3) Tumor collection location: 20 cases of upper esophagus, 35 cases of middle esophagus, 21 cases of lower esophagus; 4) Tumor size: more than 100 cm^3^; 5) Ethnicity: Han. All experiments have been approved by the Committee on the Medical Ethics of Ningbo Yinzhou NO.2 Hospital.

## Cell culture and transfection

ESCA cells EC9706, ECA109 and normal human esophageal epithelial cells SHEE were cultured in DMEM basic medium with 10% fetal bovine serum (Gibco, Gaithersburg, MD, USA) and 100 U/mL penicillin and 100 µg/mL streptomycin (Gibco) at 37°C in a humidified cell culture incubator (Thermo Fisher Scientific, Waltham, MA) with 5% CO2. All cell lines were authenticated by short tandem repeat profiling and tested for mycoplasma contamination.

EC9706 and ECA109 cells were washed twice with 1 X phosphate buffer saline (PBS), trypsinized and resuspended in fresh medium, and then seeded into cell plates according to the requirements of different experiments. SiRNA targeting lncRNA DDX11-AS1, pcDNA3.1-lncRNA DDX11-AS1 and pcDNA3.1-RBX1 overexpression plasmid, miR-514b-3p inhibitor, miR-514b-3p-mimic and their corresponding control were prepared and purchased from Shanghai Genechem Co., Ltd (Shanghai, China). Transfection of the above molecules into cells was performed using Lipofectamine 2000 (Invitrogen, California, USA) and then the corresponding assays were performed.

## Quantitative real-time PCR analysis

The RT-qPCR experiment was carried out according to the previous study [24]. Trizol reagent (Thermo Fisher Scientific) was used to extract RNA from ESCA tissues and cells according to the instructions. The extracted total RNA was dissolved in DEPC water and its concentration was measured with NanoDorp. 5 μg of total RNA was use for reverse-transcription into cDNA using a reverse transcription kit (Invitrogen). The resulted cDNA was diluted to 40ng/μL and analyzed in a 7500 Real Time PCR System (Applied Biosystems/Life Technologies, Carlsbad, CA, USA) using SYBR premix EX TAQ II kit (Takara, Dalian, China). Finally, the 2^–∆∆Ct^ method was used to analyze the real-time PCR results and GAPDH was used as the internal reference gene. All primer sequences are synthesized and purchased from Shanghai Sangon Biotechnology Co., Ltd. (Shanghai, China): lncRNA DDX11-AS1, F 5ʹ-CTGGCTACTCTTCCTCCTGG-3ʹ, R 5ʹ-CAGAGGACATGTGGGAGGTT-3ʹ; miR-514b-3p, F 5ʹ-GCAATCATGTGTAATTAGATATG-3ʹ; R 5ʹ-CTCCACTCACAG

AGGTGTC-3ʹ; RBX1, F 5ʹ-TTGTGGTTGATAACTGTGCCAT-3ʹ, R 5ʹ-GACGCCT

GGTTAGCTTGACAT-3ʹ.

## Fluorescence in situ hybridization (FISH)

RNAscope kit (Invitrogen, CA, United States) was used to perform fluorescence in situ hybridization (FISH) according to manufacturer’s instructions. Briefly, The ESCA tissue and normal tissue adjacent to the cancer were fixed with 4% paraformaldehyde and embedded in paraffin. Then sections with a thickness of 5 μm was obtained by a microtome. After the sections were deparaffinized and hydrated, the tissues were hybridized with lnc RNA DDX11-AS1 probe with Cy3 fluorescent dye (RiboBio Co. Ltd., Guangzhou, China) at 50°C in hybridization buffer for 3 hours, and then the mounting media containing DAPI (Vector Lab, Inc., Burlingame, CA, United States) was used to mount tissue sections. Finally, the expression intensity and localization of lncRNA DDX11-AS1 were observed by confocal laser microscope [25].

## Kaplan-Meier survival analysis

The median expression value of lncRNA DDX11-AS1 in ESCA patients tumor tissues was used as the cutoff value for low and high expression group classification. ESCA patients were divided into lncRNA DDX11-AS1 high expression group and low expression group. Kaplan–Meier survival curves for high and low expression group were analyzed using KM-plotter plot database (https://kmplot.com/analysis/).

## CCK-8 cell proliferation assay

EC9706 and ECA109 cells were transfected with siRNA targeting lncRNA DDX11-AS1 or its control, and co-transfected with siRNA targeting lncRNA DDX11-AS1 and miR-514b-3p inhibitor or pcDNA3.1-RBX1. 48 hours after transfection, cells were seeded in to a 96 – well plate at a density of 1500 cell/well and cultured in a humidified cell culture incubator for 0, 24, 48, 72 and 96 hours, respectively. Subsequently, 10 μL CCK8 reaction solution was added to the cell culture at indicated time point and incubated for 1 hour in a humidified cell culture incubator. The light absorption value (OD value) in each condition was captured at 450 nm wavelength on a microplate reader (Thermo Fisher Scientific) [26].

## Cell cycle detection

The DNA content (cell cycle) detection kit (Soleibao, China) was used to perform cell cycle determination according to manufacturer’s instructions. Briefly, EC9706 and ECA109 cells with different treatments collected and resuspended in staining buffer at a concentration of 1 × 10^6^ /mL. 1 mL of the cell suspension was taken out and centrifuged at 500 xg for 5 mins. The cell pellet was resuspended in cold 70% ethanol for fixation at −20°C for 2 hours. Subsequently, cells were resuspended in 100 μL RNase A solution and incubated in a 37°C water bath for 30 mins. 400 μL Propidium Iodide (PI) was added to the cell suspension and incubated at 4°C for 30 mins. The DNA contents in cells were detected by a flow cytometry (BD FACS CantoTM II Flow Cytometer) [27].

## Colony formation assay

The ESCA cells EC9706 or ECA109 with indicated treatment were trypsinized and resuspended in culture medium. Cells were seeded into a 6-well plate (1000 cells/well) and cultured for 14 days, and the culture medium was changed every 3 days during the period. After 14 days, cells were fixed with 4% paraformaldehyde at room temperature for 10 mins and stained with Giemsa reagent (Giemsa Stain Kit, Abcam ab150670) for 20 mins. Subsequently, the number of colonies was counted and the morphology of the colonies was photographed under Leica AM6000 microscope [28].

## Cell migration and invasion assay

The transwell experiment was carried out according to the previous report [29]. EC9706 and ECA109 cells with different treatments were trypsinized and resuspended in serum-free medium. The transwell upper chamber (Corning, NY, USA) without Matrigel (BD Biosciences, Bedford, MA) was used for migration assay, while transwell upper chamber coated with Matrigel was used for invasion assay. Cells were inoculated into the transwell upper chamber in serum-free medium and 500 μL of 10% serum-containing medium was added to the lower chamber. After 48 hours, culture medium was discarded and the cells were fixed with 4% paraformaldehyde at room temperature for 10 mins and stained with 0.5% crystal violet (Sigma-Aldrich, Steinheim, Germany) for 20 mins. Cells were photographed under Leica AM6000 microscope and the number of invading cells was counted.

## Apoptosis assay

EC9706 and ECA109 cells with different treatments were trypsinized and washed twice with 1XPBS, and resuspended in the staining solution. The detection of cell apoptosis was performed using the apoptosis kit (BD Biosciences, PharMingen, San Jose, CA, USA) according to the manufacturer’s instructions. In brief, 5 μL Annexin V-FITC and 5 μL PI were added to the 1000 μL cell resuspension with 1 million cells and incubated for 30 mins in the dark. Stained cells were centrifuged and washed twice with 1XPBS and resuspended in 400 μL PBS. The percentage of apoptotic cells was detected by BD FACS CantoTM II Flow Cytometer (BD Biosciences) [30,31].

## Bioinformatics analysis

The expression of lncRNAs was analyzed using the Cancer RNA-Seq Nexus database (http://syslab4.nchu.edu.tw/). Then the expression of candidate target of lncRNA DDX11-AS1 was further confirmed by GEPIA database (http://gepia.cancer-pku.cn/), based on the samples from TCGA database. The downstream targets of lncRNA DDX11-AS1 and miR-514b-3p were predicted through Starbase 2.0 database (http://starbase.sysu.edu.cn/) and TargetScan software (http://www.targetscan.org/

vert_72/), respectively.

## Dual-Luciferase reporter assay

Starbase 2.0 database and TargetScan software were used to predict the binding sites between lncRNA DDX11-AS1 and miR-514b-3p, and between miR-514b-3p and RBX1, respectively. The sequence containing the wild-type (WT) binding sites and mutation (mut) binding sites of lncRNA DDX11-AS1 and miR-514b-3p was cloned into the pmirGLO luciferase reporter plasmid vector (Promega, Madison, WI, USA) to construct recombinant pmirGLO-WT-lncRNA DDX11-AS1 and pmirGLO-mut-lncRNA DDX11-AS1 luciferase reporter plasmid. Subsequently, pmirGLO-WT-lncRNA DDX11-AS1/pmirGLO-mut-lncRNA DDX11-AS1 and miR-NC/miR-514b-3p and Renilla luciferase (Rluc) control plasmids were co-transfected into EC9706 and ECA109 cells in 24-well-plates (5X10^4^ cells/well) using Lipofectamine 2000 (Invitrogen). Similarly, the sequence containing the wild-type binding site or mutation binding site of miR-514b-3p and RBX1 was also cloned into the pmirGLO luciferase reporter plasmid vector to construct the recombinant luciferase reporter plasmid pmirGLO-WT-RBX1 or pmirGLO-mut-RBX1. The constructed luciferase reporter plasmid and miR-NC or miR-514b-3p were co-transfected into EC9706 and ECA109 cells with Rluc control plasmid. 48 hours after co-transfection, a dual luciferase reporter assay kit (Promega, Madison, WI, USA) was used to detect luciferase activity according to the manufacture’s instruction [32].

## Western blot

The western blot experiment was carried out according to the previous report [29]. The cells were lysed by cell lysis buffer on ice for half an hour and centrifuged at 10,000xg to remove cell debris. The BCA kit (Thermo Fisher Scientific) was used to quantify protein concentration. 10 µg total proteins were separated by SDS-PAGE gel and transferred to the polyvinylidene fluoride (PVDF) membrane. The PVDF membrane carrying the protein was blocked by 5% skimmed milk for one hour, and incubated with the primary antibodies: anti-RBX1 (CST, #11,922, 1:1000) and anti-GAPDH (CST, #5174, 1:1000) at 4°C overnight. After washing with TBST for 3 times, the membrane was further incubated with HRP-labeled secondary antibody (ProteinTech, SA00001-2, 1:2000) for one hour. After washing with TBST for 3 times, enhanced chemiluminescence reagent (Thermo Fisher Scientific) was used to develop the protein bands.

## Statistical analysis

SPSS 13 software (SPSS Inc., Chicago, IL, USA) was used to perform statistical calculations. GraphPad Prism 6 software was used to generate statistical graphs. The data was presented in the form of mean ± standard deviation (SD). Student’s t-test was used to compare the difference between the experimental group and the control group, and One-way analysis of variance (ANOVA) was used to compare the difference among multiple groups. Log-rank test was used to compare the survival difference between the different groups. P < 0.05 was considered as statistically significant.

## Results

Our research aims to clarify the role and potential mechanism of lncRNA DDX11-AS1 in regulating the progression of ESCA. We first analyzed the expression of lncRNA DDX11-AS1 in ESCA tissues and cell lines, and found that the expression of lncRNA DDX11-AS1 in ESCA tissues and cell lines was significantly increased, and lncRNA DDX11-AS1 also predicted a poor prognosis, so we speculated that lncRNA DDX11-AS1 was a tumor-promoting factor in regulating the progression of ESCA. Subsequently, we conducted a loss-of-function experiment in ESCA cell lines to verify the regulation of lncRNA DDX11-AS1 on the malignant phenotype of ESCA cells. Finally, we clarified the molecular mechanism of lncRNA DDX11-AS1 regulating the progression of ESCA through bioinformatics predictions and corresponding verification experiments.

## LncRNA DDX11-AS1 expression is significantly up-regulated in ESCA tissues and cell lines

**Figure 1.**
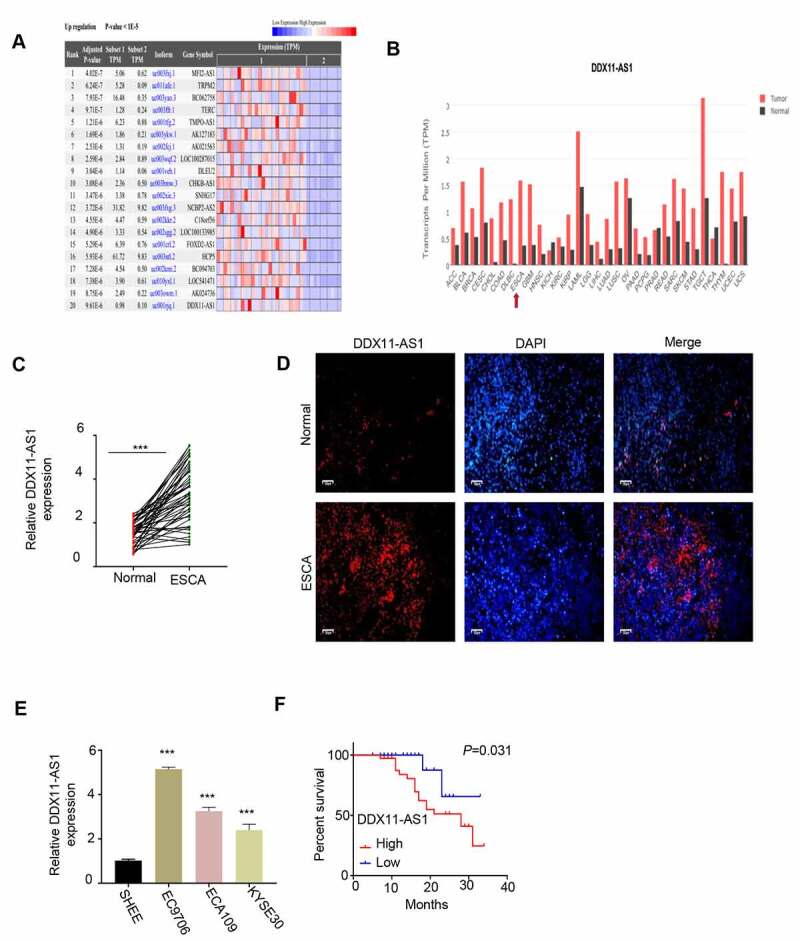
**The expression of LncRNA DDX11-AS1 was significantly increased in ESCA tissues and cell lines. A**. Analyze the expression difference of lncRNA in ESCA tissue and normal tissue adjacent to the cancer by RNA-seq data in the TCGA database. **B**. Analyze the expression difference of lnc RNA DDX11-AS1 in ESCA tissues and normal tissues adjacent to cancer by GEPIA database. **C**. The expression levels of lnc RNA DDX11-AS1 in 76 pairs of ESCA tissues and adjacent normal tissues were detected by RT-qPCR. **D**. The expression levels of lnc RNA DDX11-AS1 in ESCA tissues and adjacent normal tissues were evaluated by fluorescence in situ hybridization. Scare bare: 50 μm. **E**. The expression level of lnc RNA DDX11-AS1 in ESCA cell lines EC9706, ECA109, KYSE30 and normal human esophageal epithelial cells (SHEE) was as KM-plotter assessed by RT-qPCR. **F**. survival curve of patients with ESCA in lnc RNA DDX11-AS1 high or low expression groups was made by KM-plotter survival analysis. Three independent assays were performed in triplicate in E. *, P < 0.05, **, P < 0.01, and ***, P < 0.001. The error bars are defined as s.d

Analysis of lncRNA expression difference was performed on the RNA-seq data of esophageal cancer in the TCGA database. The analysis showed that the expression of lncRNA DDX11-AS1 in ESCA tissues was significantly higher than of normal tissues (Figure 1a). We further verified the above results through the GEPIA database and found that the lncRNA DDX11-AS1 expression in ESCA tissues was greatly increased when compared with normal tissues (Figure 1b). We also collected ESCA tumor samples and adjacent normal tissues form patients and we found that lncRNA DDX11-AS1 expression was significantly upregulated in tumor samples (Figure 1c). We further confirmed the upregulation of lncRNA DDX11-AS1 by fluorescence in situ hybridization (FISH). FISH signals of lncRNA DDX11-AS1 were much stronger in ESCA tumor samples when compared with normal tissues (Figure 1d). Lastly, we compared lncRNA DDX11-AS1 expression between ESCA cell lines EC9706, ECA109 and KYSE30 and normal human esophageal epithelial cells SHEE. qPCR analysis consistently showed that the upregulation of lncRNA DDX11-AS1 in ESCA cancer cell lines (Figure 1e). To assess whether the expression level is correlated with the revival rate, ESCA patient samples were divided into lncRNA DDX11-AS1 high and low expression group based on its median expression level in ESCA tumor samples. Kaplan-Meier survival analysis showed that a poor prognosis of patients was associated with high lncRNA DDX11-AS1 expression (figure 1f). Collective, our data suggest that lncRNA DDX11-AS1 may function to promote tumorigenesis sin ESCA cancer.

**Silencing lncRNA DDX11-AS1 inhibits ESCA cell proliferation, migration and invasion, and promotes cell apoptosis**

**Figure 2.**
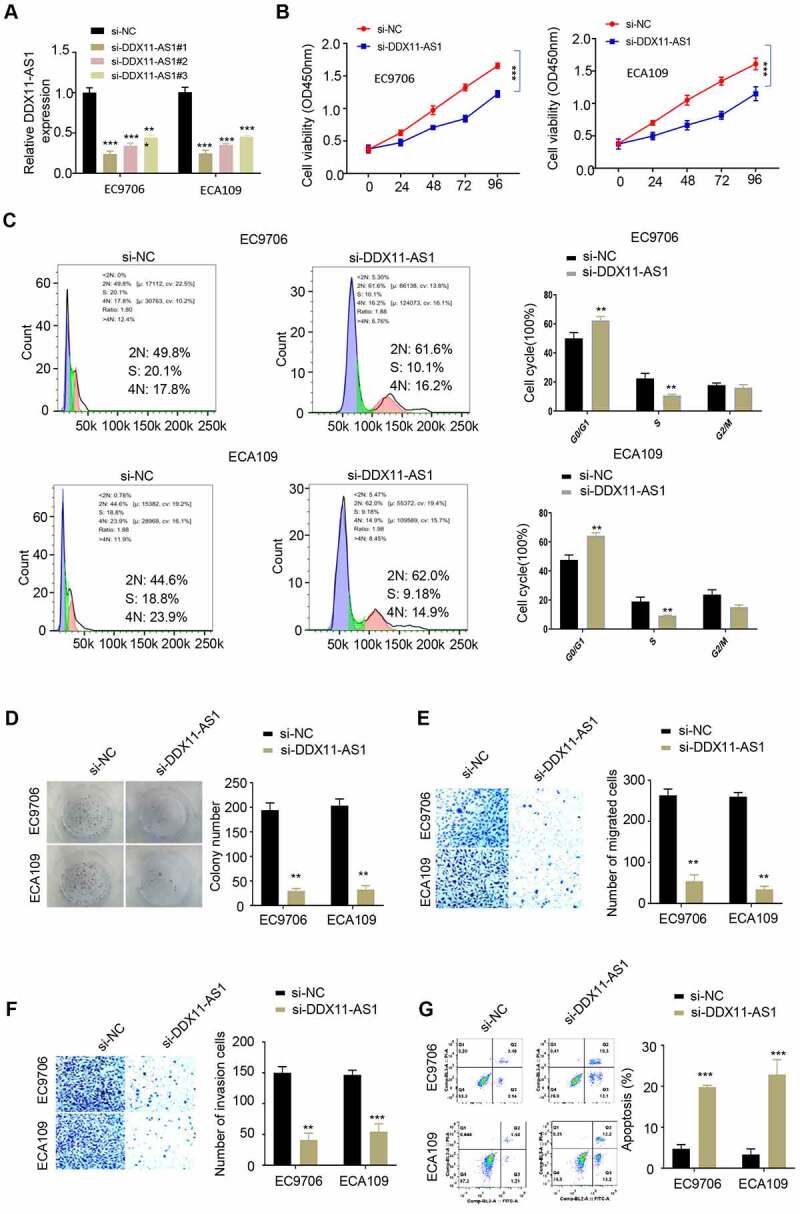
**Low expression of lncRNA DDX11-AS1 reduced the proliferation, migration and invasion of ESCA cells, and enhanced apoptosis. A**. The silencing efficiency of lnc RNA DDX11-AS1 was detected by RT-qPCR. **B**. The absorbance values of EC9706 and ECA109 cells with lnc RNA DDX11-AS1 knockdown and their control cells at 0 h, 24 h, 48 h, 72 h and 96 h were detected by CCK-8. **C**. The effect of lnc RNA DDX11-AS1 on the cell cycle was detected by fluorescence activated flow cytometry. **D**. The colony formation ability of EC9706 and ECA109 cells with lnc RNA DDX11-AS1 knockdown and their control cells were tested by colony formation assays. **E, F**. The migration and invasion abilities of EC9706 and ECA109 cells with lnc RNA DDX11-AS1 knockdown and their control cells were assessed by Transwell assay. **G**. The apoptotic status of EC9706 and ECA109 cells with lnc RNA DDX11-AS1 knockdown and their control cells was evaluated by flow cytometry. Three independent assays were performed in triplicate in the above data. *, P < 0.05, **, P < 0.01, and ***, P < 0.001. The error bars are defined as s.d

In order to validate the functional role of lncRNA DDX11-AS1 in ESCA, we selected two ESCA cell lines EC9706 and ECA109 with high level of lncRNA DDX11-AS1 expression, and knocked down lncRNA DDX11-AS1 in these cells (siControl, si – lncRNA DDX11 – AS1#1, si-lncRNA DDX11-AS1#2). The silencing efficiency was evaluated via qPCR method and both siRNAs could effectively downregulate lncRNA DDX11-AS1, with si-lncRNA DDX11-AS1#1 showing a higher silencing efficiency (Figure 2a). Subsequently, CCK-8 proliferation assay, colony formation assay, transwell migration assay, transwell invasion assay and apoptosis assay were used to detect cell proliferation, colony formation, migration, invasion capability and the percentage of apoptotic cells. Our results showed that silencing lncRNA DDX11-AS1 effectively reduced cell proliferation (Figure 2b) and arrested cell cycle progression (Figure 2c). It also impaired cell colony formation (Figure 2d), cellular migration (Figure 2e) and invasion capabilities (figure 2f). A higher level of level of cell apoptosis was also observed when lncRNA DDX11-AS1 was silenced (Figure 2g). These results further supported that lncRNA DDX11-AS1 is indispensable for malignant phenotypes of ESCA cells.

## LncRNA DDX11-AS1 acts as a sponge for miR-514b-3p

**Figure 3.**
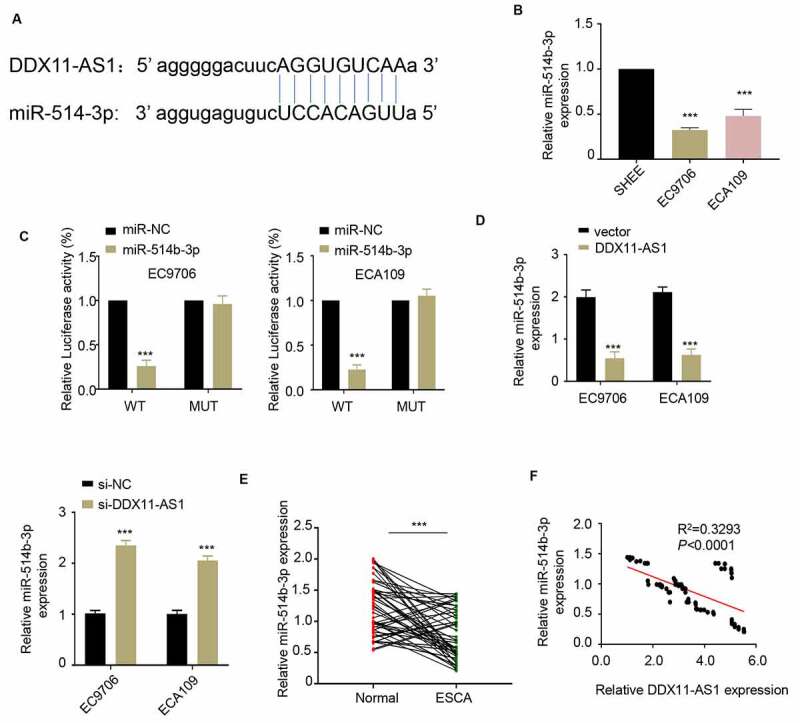
**LncRNA DDX11-AS1 competitively inhibits the expression of miR-514b-3p. A**. The potential binding sites of miR-514b-3p in lnc RNA DDX11-AS1 3ʹUTR region were analyzed by Starbase 2.0 database (http://starbase.sysu.edu.cn/). **B**. The expression of miR-514b-3p in ESCA cell lines EC9706 and ECA109 and normal human esophageal epithelial cells SHEE was detected by RT-qPCR. **C**. The binding of miR-514b-3p and lnc RNA DDX11-AS1 in EC9706 and ECA109 cells was assessed by the luciferase reporter gene assay. **D**. The expression of lnc RNA DDX11-AS1 after miR-514b-3p overexpression or silencing was evaluated in EC9706 and ECA109 cells by RT-qPCR. **E**. The expression of miR-514b-3p in 76 ESCA tissues and adjacent normal tissues was detected by RT-qPCR. **F**. The correlation between the expression of lnc RNA DDX11-AS1 and miR-514b-3p in 76 ESCA tissues was evaluated by Spearman’s correlation coefficient analysis. Three independent assays were performed in triplicate in A-E. *, P < 0.05, **, P < 0.01, and ***, P < 0.001. The error bars are defined as s.d

To explore the potential mechanism of lncRNA DDX11-AS1 in regulating ESCA progression, we predicted the downstream target molecules of lncRNA DDX11-AS1 in the Starbase 2.0 database and identified that miR-514b-3p had a binding site on lncRNA DDX11-AS1 (Figure 3a). Therefore, lncRNA DDX11-AS1 may serve as a competitive endogenous RNA (ceRNA) of miR-514b-3p. Consistently, miR-514b-3p expression in ESCA cells was significantly lower than that of normal human esophageal epithelium cells (Figure 3b). To further confirm a functional interaction, we performed luciferase reporter assay and found that the overexpression of miR-514b-3p could inhibit the luciferase activity driven by lncRNA DDX11-AS1, but the activity of mutated lncRNA DDX11-AS1 sequence was unaffected (Figure 3c). Furthermore, the overexpression of lncRNA DDX11-AS1 down-regulated the expression of miR-514b-3p, on the contrary silencing lncRNA DDX11-AS1 up-regulated the expression of miR-514b-3p. (Figure 3d). We further assessed the miR-514b-3p expression in 76 ESCA tissues and adjacent normal tissues. The miR-514b-3p expression level in ESCA tissues was largely reduced (Figure 3e). We also analyzed the relationship between the expression level of lncRNA DDX11-AS1 and miR-514b-3p in 76 ESCA tumor tissues. Spearman correlation analysis revealed that lncRNA DDX11-AS1 expression was negatively correlated with miR-514b-3p expression in tumor tissue (figure 3f). The above results collectively showed that lncRNA DDX11-AS1 seems to function as a molecular sponge to bind and absorb miR-514b-3p.

## MiR-514b-3p targets RBX1 and inhibits its expression in ESCA cells

**Figure 4.**
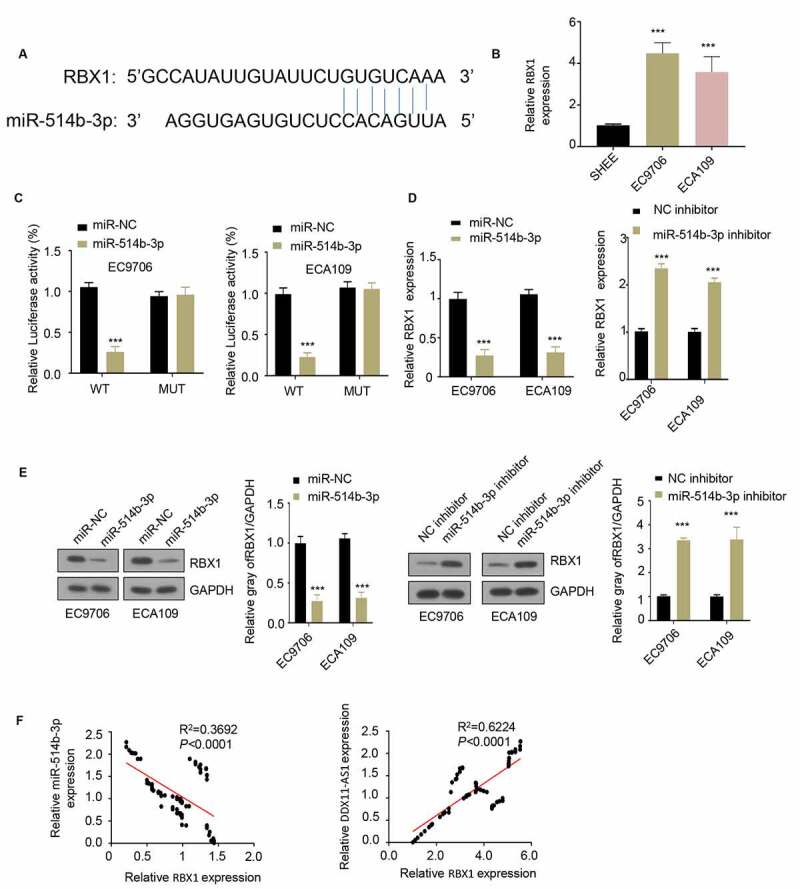
**MiR-514b-3p binds to RBX1 mRNA and inhibits its expression in ESCA cells. A**. The binding of miR-514b-3p and RBX1 3ʹ – UTR was predicted by TargetScan software (http://www.targetscan.org/vert_72/). B. The expression of RBX1 in ESCA cell lines EC9706, ECA109 and normal human esophageal epithelial cells SHEE was assessed by RT-qPCR. **C**. The binding of miR-514b-3p to RBX1 3ʹ-UTR in EC9706 and ECA109 cells was detected by the luciferase reporter gene assay. **D, E**. The expression of RBX1 mRNA and protein was assessed by RT-qPCR (d) and western blot (e) after overexpression or silencing of miR-514b-3p in EC9706 and ECA109 cells. **F**. The correlation between the expression of RBX1 and lnc RNA DDX11-AS1 or miR-514b-3p in ESCA tissues was evaluated by Spearman correlation coefficient analysis. Three independent assays were performed in triplicate in B-E. *, P < 0.05, **, P < 0.01, and ***, P < 0.001. The error bars are defined as s.d

In order to identify the downstream target of miR-514b-3p, we used TargetScan software to predict the target of miR-514b-3p and found a binding site for miR-514b-3p on RBX1 3ʹUTR (Figure 4a). The expression level of RBX1 in ESCA cells EC9706 and ECA109 was upregulated when compared with that in normal human esophageal epithelial cells SHEE (Figure 4b), suggesting a functional role of RBX1. We next applied luciferase reporter assay to assess the functional regulation of miR-514b-3p on RBX1. miR-514b-3p suppressed the luciferase activity of RNX1 3ʹUTR, however, this effect was abrogated in the mutated 3ʹUTR (Figure 4c). Additionally, the overexpression of miR-514b-3p decreased RBX1 mRNA and protein level, while silencing miR-514b-3p increased RBX1 mRNA and protein level (Figure 4d and e). Correlation analysis further showed that there was a significant positive correlation between the expression of RBX1 and lncRNA DDX11-AS1, and a negative correlation between the expression of miR-514b-3p and RBX1 (figure 4f). The above results indicate that RBX1 is a downstream target gene of miR-514b-3p, and miR-514b-3p binds to RBX1 3ʹUTR to inhibit RBX1 expression.

## MiR-514b-3p/RBX1 axis mediates the oncogenic effect of lncRNA DDX11-AS1 in ESCA cells

**Figure 5.**
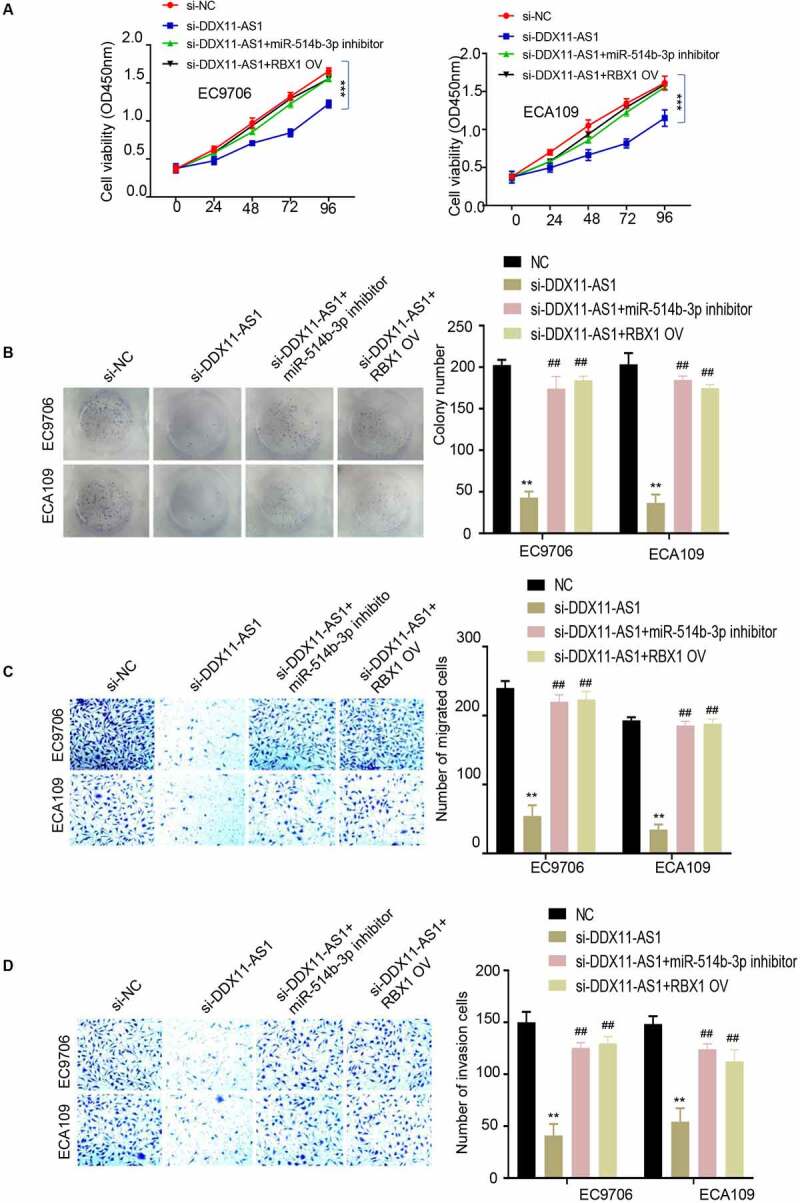
**MiR-514b-3p overexpression or RBX1 knockdown reversed the ability of lncRNA DDX11-AS1 to promote the aggressive phenotypes of ESCA cells. A**. The proliferation ability of EC9706 and ECA109 cells with indicated treatment was detected by CCK-8. **B**. The colony formation ability of EC9706 and ECA109 cells with indicated treatment was tested by the colony formation assay. **C**. The migration and invasion ability of EC9706 and ECA109 cells with indicated treatment was evaluated by Transwell (without Matrigel for migration and with Matrigel for invasion). Three independent assays were performed in triplicate in the above data. *, P < 0.05, **, P < 0.01, and ***, P < 0.001. The error bars are defined as s.d

To demonstrate the role of miR-514b-3p/RBX1 axis in the regulation of ESCA progression, EC9706 and ECA109 cells were treated under the following conditions: negative control, si-lncRNA DDX11-AS1, si-lncRNA DDX11-AS1 and miR-514b-3p inhibitor, si-lnc RNA DDX11-AS1 + RBX1 overexpression. Subsequently, CCK-8 proliferation assay, colony formation assay, transwell migration assay and invasion assay were performed. The results showed that silencing lncRNA DDX11-AS1 inhibited cell proliferation, colony formation, migration and invasion capabilities. When miR-514b-3p was inhibited or RBX1 was overexpressed, cell proliferation (Figure 5a), colony formation (Figure 5b), and migration (Figure 5c) and invasion (Figure 5d) capabilities were partially recovered. These results support the notion that MiR-514b-3p/RBX1 axis mediates the oncogenic effect of lncRNA DDX11-AS1 in ESCA cells.

## Discussion

In summary, we found that lncRNA DDX11-AS1 was highly expressed in ESCA tissues, and patients with high expression level of lncRNA DDX11-AS1 showed a poorer prognosis. This suggests that lncRNA DDX11-AS1 is a tumor-promoting factor in ESCA. After silencing lncRNA DDX11-AS1 in ESCA cells, the proliferation ability of the cells was suppressed, as well as the invasion and migration abilities, indicating that lncRNA DDX11-AS1 is required for the malignant phenotype of ESCA. Through the analysis of the Starbase 2.0 database, we found that miR-514b-3p is a downstream target molecule of lncRNA DDX11-AS1. Functional experiments confirmed that the overexpression of lncRNA DDX11-AS1 largely inhibited the expression of miR-514b-3p. Furthermore, luciferase reporter gene assay showed that miR-514b-3p inhibited the luciferase activity of the reporter plasmid containing the sequence with miR-514b-3p and lnc RNA DDX11-AS1 binding site, while the mutation in the binding site abrogated the effect. These data indicate that LncRNA DDX11-AS1 acts as a miR-514b-3p sponge and inhibits the expression of miR-514b-3p. Through TargetScan analysis, we found that RBX1 is a downstream target of miR-514b-3p, and the overexpression of miR-514b-3p repressed the expression of RBX1. Therefore, we concluded that the role of lncRNA DDX11-AS1 in promoting the development of ESCA may be mediated by sponging miR-514b-3p to sustain the expression of RBX1.

Accumulated studies have showed the functional roles of lncRNA in tumor development. For example, LncRNA MRPL23-AS1 promotes tumor progression in osteosarcoma [33], lncRNA PGM5P4-AS1 and DNAJC3-AS1 inhibit the deterioration of lung cancer [34] and colon cancer [35], respectively. Interestingly, all these lncRNA s functions by targeting miRNA-mRNA axis. lncRNA DDX11-AS1 is implicated in the development of multiple types of cancer. LncRNA DDX11-AS1 is highly expressed in GC tissues and promotes the proliferation of GC cells by inhibiting the expression of miR-873-5p to regulate signal peptidase complex 18 (SPC18) [36]. Moreover, lncRNA DDX11-AS1 is also highly expressed in hepatocellular carcinoma tissues, which is suggested as a potential marker for targeted therapy of hepatocellular carcinoma [37]. In bladder cancer and gastric cancer, lncRNA DDX11-AS1 also functions as a carcinogenic lncRNA, which promotes the progression of cancer [38,39]. There is additional evidence that lncRNA DDX11-AS1 has carcinogenic roles in ESCA, and its expression promotes paclitaxel resistance in ESCA [40]. Our study elucidated the important regulatory function of lncRNA DDX11-AS1 on the malignant phenotypes of ESCA cells and revealed the underlying mechanisms. We discovered that miR-514b-3p is a downstream regulator of lnc RNA DDX11-AS1. The sponge absorption of miR-514b-3p by lncRNA DDX11-AS1 is crucial for its function. Our findings enrich the understanding of the regulatory network by lncRNA DDX11-AS1 in ESCA.

Recent studies also suggested that RBX1 plays an oncogenic role in ESCC. RBX1 is highly expressed in ESCC and promotes the growth of cancer cells. The silencing of RBX1 induces apoptosis by upregulating the pro-apoptotic protein NOXA, which provides evidence for RBX1 as a potential drug target for ESCA [41]. Our study identified miR-135b as an upstream inhibitor of RBX1. miR-135b plays a crucial role in the progression of malignant melanoma [42]. In lung adenocarcinoma, MiR-378 and MiR-1827 also regulate the growth and invasion of cancer cells by targeting RBX1 [43]. Similarly, miR-194 regulates the growth and invasion of gastric cancer cells through RBX1 [44]. In ESCA, RBX1 seems to be a crucial regulator of a series of malignant phenotypes such as cell proliferation and invasion. This provides further evidence for RBX1 as a potential target for the treatment of ESCA.

The focus of miRNA/mRNA axis in lncRNA DDX11-AS1 regulation in ESCA was based on the evidence that miR-514b-3p has been reported in the regulation of colorectal cancer metastasis [45]. We also showed that it is a binding partner with lncRNA DDX11-AS1. On the other hand, we chose to study RBX1 since previous studies have found that RBX1 is linked with the survival in ESCA [41]. Overall our study highlighted a novel regulatory module of lncRNA DDX11-AS1/miR-514b-3p/RBX1 in supporting ESCA progression, which provides insights into the development of novel therapy by targeting lncRNA DDX11-AS1/miR-514b-3p/RBX1 axis in ESCA.

## Conclusion

This study revealed a functional role and the underlying mechanism of lncRNA DDX11-AS1 in sustaining the progression of ESCA. The upregulation of lncRNA DDX11-AS1 in ESCA tissues and cells line seems to support a more aggressive phenotype. LncRNA DDX11-AS1 promotes the proliferation, migration and invasion, and inhibits the apoptosis of ESCA cells through the miR-514b-3p/RBX1 axis. Future animal study will be required to evaluate the anti-tumor effect by targeting DDX11-AS1/miR-514b-3p/RBX1 axis in ESCA tumorigenesis.

## Highlights

1. LncRNA DDX11-AS1 is highly expressed in ESCA tissues and cell lines.
2. ESCA patients with high expression of lncRNA DDX11-AS1 have poor prognosis.
3. LncRNA DDX11-AS1 regulates the malignant phenotype of ESCA cells through miR-514b-3p/RBX1 axis.

## Data statement

Data will be provided on reasonable request.
